# An integrative model as a step toward increasing the awareness of eating disorders in the general population

**DOI:** 10.3389/fpsyt.2023.1184932

**Published:** 2023-05-03

**Authors:** Octavian Vasiliu

**Affiliations:** Department of Psychiatry, Dr. Carol Davila University Emergency Central Military Hospital, Bucharest, Romania

**Keywords:** orthorexia nervosa, eating disorder, food addiction, anorexia athletica, night eating syndrome, emotional eating, diabulimia

## Abstract

Eating disorders (EDs) represent a contradictory chapter of clinical psychiatry, i.e., although they are associated with significant prevalence and risks in the long term (including vital risk, especially for anorexia nervosa), the therapeutic resources are minimal and based on low-quality data. Another contradiction arose in the last few decades, i.e., a variety of new EDs have been described, either by clinicians or signaled by mass media, but their systematic exploration is progressing very slowly. Entities like “food addiction,” “orthorexia nervosa,” or “emotional eating disorder” still require intensive exploration in order to find the most accurate diagnostic instruments, diagnosis criteria, prevalence data, vulnerability factors, and therapeutic approaches. This article is focused on integrating into a comprehensive model a variety of EDs not specified or loosely defined by the current international classifications of psychiatric disorders. This framework is intended as an instrument for stimulating clinical and epidemiological research, with potential favorable consequences for therapeutic research. The dimensional model suggested here includes four main categories that accommodate the already recognized EDs (i.e., anorexia nervosa, bulimia nervosa, and binge eating disorder) as well as ten EDs that still need intensive research to find their clinical and pathophysiological characteristics. More good-quality studies are urgently required regarding this topic, based on the mental and physical negative impact these EDs may have in the short and long term, especially in vulnerable populations (e.g., pregnant women, athletes, adolescents, etc.).

## 1. Introduction

Eating disorders (ED) are associated with a tendency toward chronicity and severe functional impairment, high rates of comorbidity and mortality, especially for anorexia nervosa (AN) (1, 2). The worldwide lifetime prevalence of these disorders was between 2.2 and 8.4% (higher for women), while the 12-month prevalence was between 0.7 and 2.2% (3). This indicator increased between 2000 and 2018 from 3.5 to 7.8%, indicating the challenge of this type of disorder for the healthcare systems (3). However, the actual prevalence of EDs may be higher because many EDs have not yet been systematically evaluated by epidemiological research due to insufficiently validated diagnostic criteria. The main EDs currently recognized by the international classifications are AN, bulimia nervosa (BN), and binge-eating disorder (BED) (4–7). Insufficiently defined EDs have begun to attract attention from clinicians and researchers in the last two decades, although they are far from being new in the context of eating pathology. These disorders have been classified by different authors as either a stage between full EDs and recovery or a transition phase between a less severe pathology and a complete ED. (1) It is also worth to be mentioned that the COVID-19 pandemic is considered by several authors as a “wake-up call for making eating disorders a priority,” especially among adolescents and young adults (8). During this pandemic, eating behavior pathology has increased, and this signals the need for developing EDs screening and early treatment policies (8, 9).

## 2. Well-defined eating disorders-main characteristics

AN is reputedly associated with the highest mortality rate within the category of psychiatric disorders, and it affects mainly teenage girls (10). The core symptoms of AN are lower body mass index (BMI), self-induced weight loss (by dieting/purging or using appetite suppressants, diuretics, or laxatives), and distorted body image (4). Associated features are endocrine dysfunctions reflected in clinical and biological changes (4). The therapeutic resources are very few, and almost 30% of these patients will not recover (10, 11). Individual, group, and family therapy are recommended, with antidepressants and antipsychotics having limited success (10). Different interventions targeting the change of gut microbiota have been attempted, but until now, with very limited success (12, 13).

BN is characterized by the presence of repetitive episodes of binge eating and compensatory behaviors destined to avert BMI increase, consisting of self-provoked vomiting, abuse of diuretics/laxatives/fasting/extreme physical activity, etc. (6, 7). Self-perception is heavily correlated with body shape/weight (7). Antidepressants and cognitive-behavioral therapy (CBT) or interpersonal therapy are considered helpful for these patients (14–16). Fluoxetine is so far the only agent approved for the treatment of BN (17). BED consists of binge eating repetitive episodes, which are accompanied by marked distress, although the patient does not present compensatory behaviors targeting weight loss (7). Program-based self-help interventions, CBT, and pharmacotherapy are available for BED, but the quality of evidence to support these interventions is very low (15). Pharmacological approaches are also explored but insufficiently validated by clinical, good-quality data (16, 18). Lisdexamfetamine dimesylate is the only drug currently approved to treat BED (19).

## 3. Other eating disorders-general characteristics

### 3.1. Drunkorexia

The core elements are specific compensatory behaviors destined to mitigate the calorie intake due to excessive alcohol consumption (2). Also called “alcoholimia,” or “food and alcohol disturbance,” drunkorexia is observed especially in young drinkers who plan in advance their participation in excessive physical exercise or improper dieting before heavy alcohol ingestion (20). The main objective of these individuals is to be able to engage in heavy alcohol drinking, while dysfunctional eating behavior may be considered only a preemptive measure (21). The consequences of these behavioral dysfunctions for mental and physical health could be severe, i.e., nutritional deficiencies, cognitive impairment, anxious and depressive symptoms, hepatic toxicity, etc. (21, 22). It was suggested by several authors that drunkorexia may be classified as a component of the “Other specified feeding and eating disorders (OSFED)” within the latest edition of the American Psychiatric Association’s Manual of Diagnostic and Statistics (DSM) to provide insurance coverage for this pathology (23).

### 3.2. Pregorexia nervosa

EDs detected in pregnant women may lead to a significant risk for negative health effects in mothers (e.g., maternal hypertension, anemia, postpartum depression) and children (e.g., low birth weight, spontaneous abortion, microcephaly) (24, 25). For clinicians, it is, however, difficult to diagnose EDs in pregnant women based solely on routine examination, because manifestations of eating behavior pathology and physiological changes associated with pregnancy may overlap (26). Therefore, collecting a detailed anamnesis, with a focus on specific eating habits and body image distortions, especially where symptoms of EDs may be suspected, is needed in this vulnerable population. Also, the clinician is encouraged to detect more discrete ED symptoms, evaluate possible dysfunctions in daily life that may be caused by such symptoms, and determine the level of the patient’s awareness of her health problems based on behavioral cues (26).

The origins of pregorexia nervosa (PN) are found in mass media as far back as 2008 when it was used to describe pregnant women who intentionally reduce calories and increase exercise to control pregnancy weight gain (27). A high level of worry related to the risk of becoming overweight and intense preoccupations with behaviors destined to mitigate this trend toward weight or shape modifications due to a healthy pregnancy is considered central to this diagnosis (27).

### 3.3. Orthorexia nervosa

This disorder is defined by obsessive thoughts about “healthy” meals and a lack of flexibility regarding the composition of daily meals, which lead to clinically significant medical or psychological impairments (28). Steven Bratman launched the concept of “orthorexia nervosa” (ON) in the late 90s to delineate the pathological preoccupation with “healthy food” or strict dietary rules (29).

The definition of ON has changed in time, and new elements have been added: the patients’ focus is on the avoidance of becoming ill, and the correspondent behavior is excessive dieting; several specific categories of foods are avoided (e.g., those rich in carbohydrates, saturated fats, artificially flavored, etc.); malnutrition or certain nutritional deficits may be detected as a consequence of restricted food ingestion; social isolation, high levels of fears and worries related to eating (30). Unlike “healthy orthorexia,” defined by a constructive interest in eating healthy foods, ON is associated with mental and physical negative consequences.

It is challenging to include ON between the OSFEDs or the obsessive–compulsive spectrum disorders because there are no unanimously accepted diagnostic criteria or sound epidemiological data (31). An online survey (*N* = 343 Italian healthcare professionals) showed that ON is considered a variant of ED by most responders, while others classified this pathology as a prodromal phase or a stage in the evolution of anorexia nervosa (AN) (32). Most responders (over 80%) favored the inclusion of ON diagnosis in the Diagnostic and Statistical Manual of Mental Disorders (DSM) classification within the category of EDs (32).

### 3.4. Muscle dysmorphia or bigorexia nervosa

This condition affects male bodybuilders, who continuously ruminate about their body mass and aspect and consider they should be larger or more muscular (33, 34). If obsessions are clearly related to the inadequacy of their body shape, compulsions refer to overexercising in the gym, overbuying sports supplements, dysfunctional eating behavior, or substance use disorders (SUD) (33). Muscle dysmorphia is synonymous with “reverse anorexia,” first described in a study that included 108 bodybuilders (34). Also, the term “Adonis complex” has been vehiculated about an excessive focus on men’s body image, based on the name of the Greek god who represented a standard of masculinity (35).

However, based on the DSM-5 criteria, muscle dysmorphia is a subtype of body dysmorphic disorder, part of the obsessive–compulsive spectrum (7). This classification of bigorexia nervosa can be seriously challenged, based on the shared clinical features with individuals diagnosed with AN and on the fact that almost one of each five patients had a history of AN, while almost one out of three had a past diagnosis of any ED (36, 37). These individuals consider themselves small and weak, although the reality is completely different, and tend to avoid social gatherings due to fears they would be seen as too fragile (34).

### 3.5. Night eating disorder

Originally described by Stunkard et al. in 1955 and associated with periods of weight gain and life stress (38), night eating syndrome (NES) is still an elusive entity with debated diagnostic criteria. In the original paper, NES was described as consuming large meals during the evening and night (≥25% of the daily caloric intake is distributed in these periods), combined with sleepiness and morning anorexia (38, 39). These manifestations have been considered secondary to a lag in the circadian distribution of food intake (39). Unlike other EDs, there are no compensatory behaviors and the episodes of night eating are not similar to the binge episodes because of the lower quantity of food ingested (38, 39). Although patients may become obese or overweight, this is not a rule (38, 39). NES is characterized by full awareness of nocturnal meals, unlike sleep-related disorders with automatic eating behaviors (38, 39).

The DSM-5 classification mentions NES in a residual category and requires for its diagnosis the existence of repetitive episodes of night eating (during awakenings or after dinner), the complete consciousness during these episodes, and the patient’s ability to remember them. There are no other confounding factors (e.g., sleep–wake cycle changes or socio-cultural norms, organic/toxic/psychiatric pathology), but there are dysfunctions and/or distress due to this problematic behavior (7).

### 3.6. Sleep-related eating disorder

This clinical entity is included in the category of “parasomnias,” and its onset is during non-REM sleep. The core elements consist in preparing and consuming food during sleep, with no memory of these behaviors when the individual wake up. Sleep-related eating disorder (SRED) is considered a “disorder of arousal,” together with sleepwalking, sexsomnia, sleep terrors, confusional arousals, and sleep-related choking syndrome (40). Unlike individuals with NES, these patients are not aware of what and when they are eating; therefore they do not remember such episodes. Several authors consider this condition represents a non-motor cluster of symptoms belonging to the restless legs syndrome (41).

For the diagnosis of SRED, the following criteria have been formulated: frequent episodes of nighttime eating with onset after sleep initiation; consumption of inedible foods or combinations of comestible and peculiar foods; potentially dangerous behaviors during sleep that are related to obtaining or preparing food; adverse health effects due to the abnormal eating behavior; either partial or complete lack of awareness regarding these episodes; there is no other condition, pharmacological, organic or psychiatric, that could explain this behavior (42, 43).

### 3.7. Emotional eating disorder

Stress, depression, irritability, and anxiety have been associated with a significant impact on eating behaviors (44). The rise of food intake behaviors as a direct reaction to negative emotional stimuli, with a focus on hyper-palatable foods, was termed “emotional eating” (EE) (44). This type of eating behavior may be considered a coping mechanism for stressful internal or external events, leading to weight increases. However, this behavior is not necessarily associated with higher body weight or obesity (44, 45). Patients with EE may frequently report concerns about their weight and dysfunctions in their body representation/general health perception and may consider EE as an acquired coping strategy they cannot control (45).

There are still doubts if EE stands for a specific phenomenon or if it is just a consequence of other EDs, and several theories have been elaborated in an attempt to explain the pathophysiology of this behavior (46). In healthy individuals, naturalistic studies based on sequential evaluation of negative emotions and eating behaviors led to contradictory results regarding the stability of the EE construct (46). However, in a study that enrolled 127 normal-weight women that explored the relationships between EE, bulimic behaviors, and restrained eating, researchers reported EE might be responsible for excessive food ingestion, and it may also be associated with general psychopathological symptoms met in EDs and chronic dieting (47). Also, individuals with significant overeating behaviors, e.g., BED, presented a consistent correlation between unpleasant emotions and food ingestion, suggesting possible classical conditioning through repeated pairing (46).

### 3.8. Food addiction

Food addiction is also known as “eating addiction,” and it is focused on the dependence on certain types of hyper-palatable foods (48). Neurobiological changes related to the reward system, preoccupation related to specific substances, impaired control of eating, social dysfunctions, risky use of a substance (e.g., hyper-palatable foods), tolerance/withdrawal, chronic evolution, and high risk of relapse have been found as key diagnostic elements of food addiction (49). Also, genetic vulnerability, substance sensitization, cross-sensitization, and impulsivity are supported by evidence as presenting a pathophysiological role in animal models of food addiction, but also in human studies (49).

Because no unanimously accepted diagnostic criteria exist for this disorder, it is difficult to interpret the results of epidemiological studies exploring food addiction and it is even more challenging to design trials with therapeutic goals. The excessive consumption of specific foods, usually hyper-palatable, rich in saturated fats, hydrocarbonates, and artificial flavors, that follows a pattern resembling the addictive behavior in SUD is considered the common ground of food addiction (48, 50).

### 3.9. Anorexia Athletica

Body image is essential for many athletes, and in several sports, low body weight may represent an advantage over one’s opponents (51). The obsessive focus on leanness and thinness in athletes may lead to anorexia if they decrease their calorie intake severely and/or tend to resort to excessive physical exercise to achieve or maintain a low body mass (51). Athletes may have lower than expected body weights and fat mass, even without presenting AA; therefore, more accurate instruments for detecting this disorder are needed, except for anthropometric parameters (51).

There are no well-defined and unanimously-accepted diagnostic criteria for AA. Still, several characteristics have been reported: the loss of body weight is due to sport/performance-related concerns, not to the appearance/body shape worries, except for cases where the degree of fatness may be related to lower chances of being successful in the respective sport; the beginning of restricting eating or excessive exercising is self-imposed or recommended by the coaching team, as part of the regular training; frequent weight cycling although some athletes may preserve a very low BMI on long-term (51).

### 3.10. Diabulimia

The co-existence of type 1 diabetes mellitus (T1DM) and an ED has been termed “diabulimia,” and the core belief of these patients is that decreasing the daily dose of insulin is needed for losing weight or maintaining their current body weight (52). This disorder is also called “type 1 disordered eating” and has been described in children, adolescents, and adults (53). Although diabulimia has only recently been systematically explored, its history is quite long, with the first cases of T1DM and comorbid ED being described more than four decades ago (54).

The risk of severe acute (e.g., diabetic ketoacidosis) or long-term (e.g., retinopathy) complications due to irregular or constantly lower insulin doses are evident in these patients; therefore, psychoeducational and therapeutic interventions should follow the detection of vulnerability factors. Because of the irregular insulin administration and disordered eating behavior, diabulimics have a 3-fold higher risk for death than non-diabetic individuals, based on an 11-year follow-up study in female patients with T1DM and a history of insulin restriction detected during the enrollment visit (55).

## 4. Discussion

**Figure 1:**
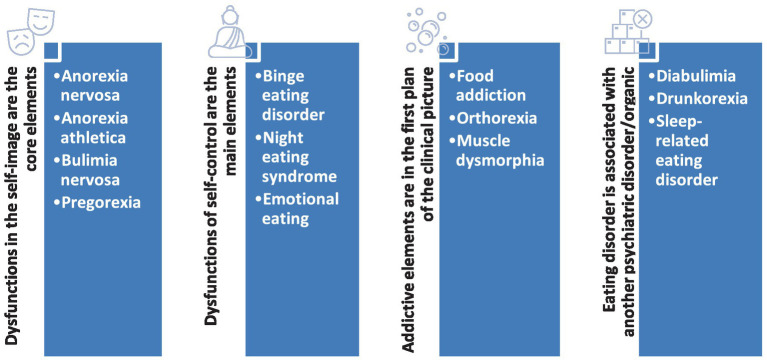
Dimensional model of eating disorders.

A review of the current data available in the literature for less well-defined EDs was considered necessary due to the need to increase the awareness of the general population about their existence (Table 1). An integrative, four-category dimensional model for EDs has been elaborated based on the limited available data (Figure 1).

**Table 1:** Main results of the review targeting insufficiently recognized EDs.

Eating disorder	Core clinical manifestations	Diagnostic criteria	Available structured instruments for evaluation	Risk factors	Epidemiology	Treatments
Drunkorexia	Excessive alcohol use + compensatory eating behavior (reduced calorie intake)	Yes (23)	Yes (56, 57)	Personal history of SUD or ED; heavy calorie restrictors; excitement seeking ↑, body esteem ↓ (21, 58–60)	Prevalence of 14–25% in the student population for drunkorexia-like behaviors (58, 61)	No RCT or OLT avaialble. Harm-reduction programs are available, but data regarding their efficacy is insufficient (61). Prevention strategies are also not yet sufficiently validated (62).
Pregorexia nervosa	Calorie intake reduction and/or excessive physical exercise in order to present the body weight during pregnancy	Yes, but loosely defined (24, 25, 63)	No, only general instruments for EDs	Dysfunctions of body image, personal history of EDs (24, 25, 63, 64)	Prevalence of 0.6–27.8% for any ED during pregnancy; for pregorexia, few data are available, but an estimation of 5% has been suggested (24, 26, 63, 64)	No good-quality data are available. Nutritional and psychosocial interventions have been recommended (25). A multidisciplinary team is advisable (25). Prevention through psychoeducational measures has been suggested in pregnant women with risk factors (35).
Orthorexia nervosa	Obsessive focus on “healthy foods,” with functional impairments	Yes (65, 66)	Yes (66–68)	Perfectionism, OC traits, history of EDs, dieting, poor body image, drive for thinness, disordered eating, higher economic status, academic professionals, anxiety, low self-esteem, and possible vegan/vegetarian diet (28, 69–72).	Very high variability in data regarding orthorexia nervosa prevalence. The most probable value is 6.9%, with higher values in certain professionals (up to 58%) (69–74).	No good-quality data are available. Treatment interventions are explored only in case reports (72–76). Prevention is advisable, but guidelines for this are lacking.
Muscle dysmorphia	Addiction to own body image or continuous ruminations about the need to be more muscular/larger	No	Yes (77, 78)	The use of steroids for bodybuilding, history of EDs (anorexia or bulimia nervosa), certain sportive categories (weightlifters, bodybuilders), and students to Exercise and Sport Sciences (33, 37)	The lifetime prevalence was between 13.6 and 44% in male weightlifters (79). In bodybuilders, the prevalence was estimated at 8.3% (34). In military personnel, the prevalence was 5–15% (80).	Psychotherapy, mainly CBT, is recommended in these patients (81). Family therapy may also be recommended (82).
Night eating disorder	High-calorie intake during nighttime + sleepiness + morning lack of appetite for food	Yes (7, 38, 83)	Yes (83–85)	Obesity/overweight, major depression, low self-esteem, adolescents with depressive/eating/sleeping pathology, and higher levels of anxiety (85–87).	In bariatric surgery samples, the prevalence was 2–20%, and in the general population 1.1% (86, 87)	CBT, psychoeducation, relaxation techniques, antidepressants, topiramate, or melatonergic agents may be recommended based on limited evidence (88–90).
Sleep-related eating disorder	Preparation of meals and eating them during sleep + lack of awareness about this behavior	Yes (42, 43)	Yes (91)	Pharmacological triggers have been identified (42, 92–94). Comorbid sleep disorders, smoking cessation, work-related stress, and weight gain/obesity (42, 91).	The estimated prevalence in vulnerable patients was 4–5% (91). Higher prevalence in hospitalized patients vs. controls (91).	Prevention and treatment of risk factors is the most supported intervention (40). No therapeutic intervention has long-term efficacy (95). Antidepressants, clonazepam, and topiramate may have limited success (96). Pramipexole may be recommended when restless legs syndrome is comorbid (96).
Emotional eating disorder	High food intake triggered by negative emotions + preference for hyper-palatable foods	No	Yes (97, 98)	Shorter sleep duration in adults, depressive symptoms, lower quality of social support, increased binge eating, and reduced self-monitoring (44, 45, 99). Very restrictive diet, low level of interoceptive insight, alexithymia, poor emotional control, inadequate parenting, and HPAA dysfunction (100). Lower emotional regulation scores in normal-eating teens (101). Female gender, non-Hispanic white, impatience, and younger age (102). Body satisfaction, eating-related self-efficacy, and self-regulating eating (103).	The estimated prevalence is highly variable in the literature (4.5 to 20.5%) (102, 104, 105).	Psychotherapy may be useful, but data are limited (105). Physical exercise programs, psychoeducation, CBT (106). Mindfulness meditation techniques may improve emotional eating and binge eating (107).
Food addiction	Dependence on certain types of hyper-palatable foods	No	Yes (108)	Negative life events during early childhood and adolescence, caloric restriction, female gender, obesity/overweight (controversial), BN, and BED (109–112).	The prevalence was estimated to vary between 5.4 and 56.8% (110–112).	A multidisciplinary team is appropriate (48). A 12-step online/by phone/face-to-face approach may be recommended (113). CBT focused on cognitive restructuring, emotion management, and normalization has been suggested (56). Brief psychoeducational interventions have been associated with favorable results (111, 112). No RCT exploring the efficacy of any pharmacological agent exists.
Anorexia athletica	Excessive focus on leanness and thinness in athletes + calorie restriction and/or overexercise	No	Yes (114)	Certain sportives or professionals (e.g., athletes, ballet dancers), prolonged periods of dieting, frequent weight variations, traumatic events, and increases in training schedule (115, 116). Perfectionism, intense desire to lose weight due to external influences, engagement in training early during life, overtraining, and negative coaching behaviors (117). Low levels of leptins (117–119).	The prevalence of EDs in athletes has been estimated to be between 4 and 19% (118, 119).	A multidisciplinary team is recommended (82). Nutritional therapy, individual CBT, family therapy, or psychological counseling may be beneficial (120).
Diabulimia	Type 1 diabetes mellitus + the belief that decreasing the dose of insulin will lead to weight loss, despite all possible risks for mental/physical health	No	No, but general scales for EDs may be used	Women, adolescents, borderline personality disorder (55, 121, 122).	Almost 20% of women with T1DM might develop diabulimia (55). A more conservative evaluation suggests a value of 7.3% for this parameter (123). EDs are more frequent in individuals with diabetes vs. the general population (124).	Psychoeducational interventions, nutritional advice, and frequent monitoring of biological parameters in vulnerable populations (89, 125). A multidisciplinary approach is recommended (55, 88, 89).

BED, binge eating disorder; BN, bulimia nervosa; CBT, cognitive behavioral therapy; D1TM, type 1 diabetes mellitus; ED, eating disorder; HPAA, hypothalamic–pituitary–adrenal axis; OC, obsessive–compulsive; OLT, open-label trial; RCT, randomized clinical trial; SUD, substance use disorder.

This perspective is based on the core elements of each nosographic category: (1) *dysfunctions in the self-image*, mainly body shape and weight, are shared by patients with anorexia nervosa, bulimia nervosa, anorexia athletica (also called “reverse anorexia”), and pregorexia; (2) *dysfunctions of the self-control*, or lack of control over their own impulses, are present in the first line of clinical manifestations in patients with BED, NES, and EE; (3) an *addictive component* is reflected by patients behaviors toward eating: food addiction (where the focus of attention is represented by hyper-palatable foods), orthorexia nervosa (the focus of attention is represented by “healthy foods”), and muscle dysmorphia (the focus of attention is own body); (4) there is *an association of* a*bnormal eating behavior and an organic pathology* (type I diabetes, in diabulimia), or *a psychiatric disorder* (alcohol use disorder, in drunkorexia, or a parasomnia, in sleep-related eating disorder).

Each category previously presented shares compensatory behaviors for the main clinical element identified as the core dysfunction. The behaviors are activated to maintain an idealized self-image for the first category, to compensate for the lack of control through excessive eating in the second category, to gather the desired resources and to derive pleasure after consuming them in the third case, and to make up for possible biological or psychological self-perceived threats, in the fourth category.

The efficacy of therapeutic interventions in EDs is still inconclusive. However, during psychotherapy, the core beliefs referring to self-image distortions may be targeted, and techniques like mindfulness, relaxation training, increasing awareness about own impulses, and psychoeducation can lead to favorable effects. Multidisciplinary approaches have been recommended for most EDs (31, 52, 88, 120, 126). While nutritional and psychoeducation interventions are frequently suggested for EDs, very few therapeutic-specific recommendations have been identified: CBT or family therapy for muscle dysmorphia, CBT, relaxation techniques, and pharmacotherapy (antidepressants, topiramate, melatonergic agents) for NES; antidepressants, clonazepam or topiramate for SRED; mindfulness, physical exercise program, or CBT for EE;12-step approach or CBT for food addiction; CBT or family therapy for AA (55, 81, 82, 88–90, 96, 106, 107, 113).

The limitations of this model are (1) lack of pathophysiological evidence for each disorder; (2) large-scale confirmatory epidemiological studies are needed, and this model may be modified by further data; (3) there is a certain degree of overlap between clinical entities because several of their main symptoms are similar (e.g., self-image dysfunctions and addictive features are equally important in muscle dysmorphia).

In conclusion, the current model, although still in its early conceptualization phase, may present pragmatic and theoretical benefits, e.g., conducting epidemiological and clinical trials, finding therapeutic approaches based on common clinical features, and enhancement of public awareness about their consequences on the general health consequences.

## Data availability statement

The original contributions presented in the study are included in the article/supplementary material, further inquiries can be directed to the corresponding author.

## Author contributions

The author confirms being the sole contributor of this work and has approved it for publication.

## Conflict of interest

The author declares that the research was conducted in the absence of any commercial or financial relationships that could be construed as a potential conflict of interest.

## Publisher’s note

All claims expressed in this article are solely those of the authors and do not necessarily represent those of their affiliated organizations, or those of the publisher, the editors and the reviewers. Any product that may be evaluated in this article, or claim that may be made by its manufacturer, is not guaranteed or endorsed by the publisher.
